# Selective logging weakly influences species co‐occurrence in a community of tropical understorey birds

**DOI:** 10.1111/1365-2656.70085

**Published:** 2025-07-08

**Authors:** David Costantini, Simone Messina, Julia Weiss, Suzanne Tomassi, Cindy C. P. Cosset, Suzan Benedick, Bruno Bellisario, David P. Edwards

**Affiliations:** ^1^ Department of Ecological and Biological Sciences University of Tuscia Viterbo Italy; ^2^ Department of Plant Sciences and Centre for Global Wood Security University of Cambridge Cambridge UK; ^3^ Conservation Research Institute, University of Cambridge Cambridge UK; ^4^ Department of Animal and Plant Sciences University of Sheffield Sheffield UK; ^5^ Wildlife Ecology and Conservation Department IFAS, University of Florida Gainesville Florida USA; ^6^ The Biodiversity Consultancy Cambridge UK; ^7^ Faculty of Sustainable Agriculture Universiti Malaysia Sabah Sandakan Sabah Malaysia

**Keywords:** avian community, co‐occurrence, environmental filtering, niche segregation, resilience, tropical forest

## Abstract

1. Selective logging is a major driver of tropical land‐use change, causing reductions in forest specialist species with concurrent increases in edge‐tolerant species. A key question is understanding how selective logging impacts co‐occurrence and assembly mechanisms in vertebrate communities as forests recover post‐logging.
2. Using a 10‐year, repeat‐sample study of understorey bird species in Borneo, we compare the structure of species co‐occurrences over time between old‐growth unlogged and logged forests, investigating the roles of functional traits and local abundance in driving co‐occurrence patterns.
3. Co‐occurrence patterns were resilient to selective logging over time, although patterns were not consistent across all species in both forest types. Species with more specialised diets showed a significant tendency towards low fidelity, while species that engage in aerial foraging, soaring and gliding exhibited a significant tendency to have low values of fidelity in both types of forest. Changes in co‐occurrence patterns were also significantly influenced by changes in local abundance.
4. Our results indicate that niche segregation and environmental filtering operate to shape the assemblage of the avian community in both forest types, but co‐occurrence was resilient to selective logging over time. Our results also underscore the role of some species in regulating avian assemblages and the long‐term conservation value of logged tropical forests.

## Graphical abstract

Our findings reveal a notable resilience in the co‐occurrence patterns of understorey birds to selective logging in Borneo, underscoring the long‐term conservation value of logged tropical forests.

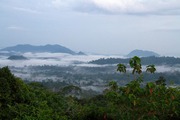

## 1 INTRODUCTION

Selectively logged forests are becoming the prevailing vegetation cover in much of the tropical biome (Malhi et al., 2014), with over 400 million hectares (~25%) of remaining old‐growth tropical forests designated for selective logging (Blaser et al., 2011; Edwards, Tobias, et al., 2014; Edwards et al., 2019). Compared to old‐growth forests, selectively logged forests are less heterogeneous, have a lower canopy often interrupted by forest gaps and have denser understorey vegetation dominated by fast‐growing pioneer species and elevated abundance of lianas (Cerullo & Edwards, 2019; Hardwick et al., 2015; Imai et al., 2019; Senior et al., 2018). Despite these changes in habitat structure, selectively logged forests can retain high levels of diversity, driven by reductions in forest‐specialist species and concurrent increases in edge‐tolerant species (Arbainsyah et al., 2014; Edwards et al., 2011; Ewers et al., 2015; Gibson et al., 2011; Malhi et al., 2022; Putz et al., 2012; Sodhi et al., 2010). A key question is understanding how selective logging impacts patterns of species co‐occurrence—the number of times where two species are found together, across all possible sets of species in the community. Addressing this gap is important to provide a mechanistic understanding of the processes that shape animal assemblages after logging disturbance.

There is solid evidence that non‐random patterns of species co‐occurrence play an important role in structuring biological communities (Diamond, 1975). Trophic niche overlap and biotic interactions (e.g. competition, mutualism, predation) are important ecological factors that regulate species co‐occurrence (Brambilla et al., 2020; Wearn et al., 2019; Wisz et al., 2013). As a result, some species co‐occur more or less often than expected by chance. This implies that increases, declines or even extinctions of given species might indirectly influence the local abundance of other species that co‐occur or avoid it (Burkle et al., 2013). Competition for resources might be important in driving the nature of co‐occurrence between species with similar trophic habits. For example, frugivore–granivore mammalian species positively co‐occurred in forests when resources were plentiful, but patterns of co‐occurrence disappeared when food availability was lower (Williams et al., 2022).

A common symptom of anthropogenic landscape transformation is the decline in abundance or spatial occupancy of some species, allowing portions of the habitat to be increasingly available to other species, driving changes in patterns of species co‐occurrence and associations (Tulloch et al., 2018). Species co‐occurrence may inform predictions of how communities respond to anthropogenic‐induced change and enable identification of mechanisms that govern the reshaping of community structures (Tulloch et al., 2018). For example, habitat alteration could promote co‐occurrence between previously non‐co‐occurring species or, conversely, the disruption of previous species co‐occurrences (Bar‐Massada & Belmaker, 2017; Gilbert et al., 2022; Larsen & Ormerod, 2014; O'Brien et al., 2018). Such patterns arise because species may shift their patterns of habitat use, for example, owing to niche partitioning. The extent to which any changes in co‐occurrence are linked to specific functional characteristics (e.g. behavioural and morphological foraging traits) of species might help elucidate which portion of functional space could be eroded by habitat loss or facilitated in habitat recovery.

Analysis of co‐occurrence networks is one analytical approach that enables the estimates of the degree of displacement or co‐occurrence between species in a given community under different circumstances (Gotelli & McCabe, 2006). For example, if species X interacts negatively with species Y, we would expect that any increase in species X would result in a decline of species Y. In case the two species X and Y show a positive co‐occurrence, we would expect a positive covariation in the local abundance of the two species. Although shared co‐occurrences cannot be considered as surrogates of real interactions between species, they help elucidate the mechanisms that might shape the assemblage of species (Blanchet et al., 2020). For example, they might reveal successions of spatial and/or temporal patterns corresponding to changes in habitat occupancy that can be attributed to specific events (Kay et al., 2018; Tulloch et al., 2018). This co‐occurrence network approach enables the identification of nuanced changes in the structure of a given community, even though the overall richness of species might not change. In addition, the quantification of the temporal changes in both network‐ and node‐level metrics could help predict the trajectories of change in pristine, disturbed and recovering habitats.

There are key knowledge gaps in our understanding of how shifts in the drivers of community assembly following habitat change in turn cause shifts in patterns of species co‐occurrence in the tropics. Species vary considerably in their resilience (ability to recover from disturbances) to selective logging; these differences are often driven by variation in species' functional traits, which are measurable characteristics of organisms that influence their performance (e.g. Costantini et al., 2016). This underscores the importance of providing species‐level assessments for improving our ecological understanding of the resilience of the whole community (e.g. maintenance of species co‐occurrence patterns) for enhancing conservation effectiveness. Previously, Edwards et al. (2021) investigated community assembly patterns in Bornean birds. They found trait convergence across primary and logged forests in both specialists and generalists, suggesting that environmental filtering—the process by which environmental conditions (e.g. abiotic conditions, habitat structural characteristics) determine which species can survive and persist in a given area—shapes avian communities after logging. However, this study was temporally limited and did not assess the responses of individual species to such changes. Addressing the long‐term temporal dynamics of the structure of avian communities is important because there may be a time lag for the appearance of more severe logging impacts on the avian community, or alternatively logging outcomes might diminish towards unlogged, old‐growth patterns.

We present a long‐term study (2014–2023) of tropical understorey birds sampled across unlogged and logged forests in Borneo to tackle four key objectives: (1) determine whether and how unlogged and logged forests differ in the structure of species co‐occurrences over time; (2) examine the tendency of a given bird species to co‐occur with other species across time; (3) quantify whether the patterns of species co‐occurrences are explained by the functional traits of species; and (4) determine the extent to which changes in the temporal patterns of co‐occurrence are driven by changes in local abundance.

## 2 MATERIALS AND METHODS

### 2.1 Study area and data collection

**FIGURE 1:**
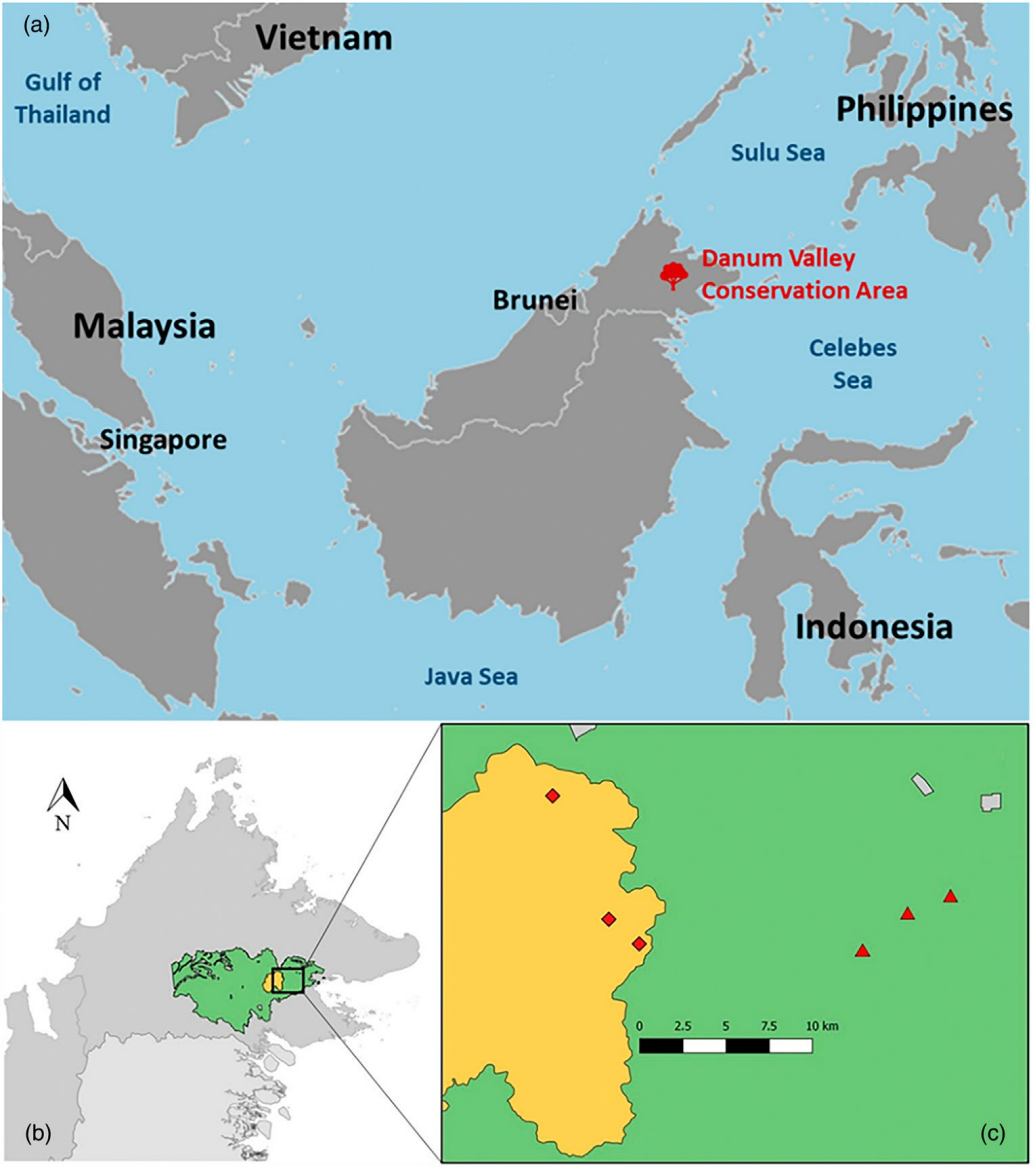
(a) Danum Valley Conservation Area in the Malaysian state of Sabah, Borneo. (b) The green area corresponds to the Yayasan Sabah logging concession, and the yellow area corresponds to the Danum Valley Conservation Area and Palum Tambun watershed reserve. (c) Distribution of study plots between unlogged forest (square symbols) and selectively logged forest (triangular symbols); grey areas are plantations. This image was previously published in Messina et al. (2020).

We carried out the fieldwork in Danum Valley Conservation Area, Palum Tambun Watershed Reserve and Ulu Segama‐Malua Forest Reserve, all within the Yayasan Sabah logging concession in Malaysian Borneo (Figure 1) from early June to late August in the years 2014–2019 and 2022–2023. Fieldwork was not authorised in 2020 and 2021 due to the COVID‐19 pandemic. The Yayasan Sabah logging concession is an ~900,000 ha contiguous and extensive lowland dipterocarp forest. We focused our sampling on the 45,200 ha unlogged old‐growth forest within the Danum Valley Conservation Area and Palum Tambun Watershed Reserve (4°57045.2″ N, 117°48010.4″ E) and selectively logged forest within the 238,000 ha of the Ulu Segama‐Malua Forest Reserve (4°57042.8″ N, 117°56051.7″ E).

We collected data on the local abundance of 95 bird species (8367 captures, available from Figshare at https://figshare.com/account/articles/29163536?file=54877529) in three unlogged old‐growth forest plots and in three forest plots. The forest was logged in the period 1970–1990 at rate of timber removal of ~113 m^3^ ha^−1^ and again in 2001–2007 at a rate of timber removal of 31 m^3^ ha^−1^ (Fisher et al., 2011) and was not subjected to any type of restoration intervention. The distance between the surveyed unlogged and logged forest plots was >10 km (Figure 1). This distance ensured negligible or absence of influence of juvenile dispersal between forest types on abundance estimates as supported by the lack of recaptures between forest types (Cosset et al., 2021). Plots were at least 1.8 km apart (mean unlogged forest = 6.64 km; mean logged forest = 4.04 km) and 500 m from the nearest road to avoid edge effects. Within each plot, we had three (two only in 2014) parallel transects, spaced at 250 m intervals, containing 15 nets (12 × 2.7 m; 25‐mm mesh size) erected end‐to‐end, run simultaneously from 06:00 to 12:00 h. The distance between transects made them statistically independent from each other (Hill & Hamer, 2004). Moreover, our transects were representative of the different environmental conditions found in unlogged and selectively logged forests. In particular, logged forests contained more saplings, fewer large trees and less vegetation cover in both the understorey and canopy than unlogged forests (Senior et al., 2018).

We visited each plot three times (twice only in 2022) per field season (estimated 1944 mist‐net hours in total) following a rotation among plots to minimise potential within‐year temporal effects. We marked every captured bird with an individual numbered ring and recorded the species. Since we carried out mist netting in the understorey, we consider all study species as part of the understorey avian community. Bird species that were captured only once were not included in this study. Recaptured individuals were included in the abundance counts because recapture rates were low (mean recapture rate across species: 0.16 individuals) and similar between unlogged and logged forests (mean frequencies difference: 0.01). We preferred to do so to avoid any arbitrary removal and because prior work showed that abundance estimates were unchanged if recaptured individuals were included (Messina et al., 2020).

### 2.2 Functional traits

The pattern and change of species co‐occurrence may be influenced by specific functional traits associated with habitat use. We used the AVONET database (Tobias et al., 2022) and EltonTraits 1.0 (Wilman et al., 2014) to select four main traits available for all our species. Specifically, we used the (i) trophic niche and (ii) foraging strata of the species, which play relevant roles in the response of understorey birds to forest logging in our study region (Messina et al., 2021, 2022). Trophic niche categories were *invertivores* for those species that depend on invertebrates for more than 50% of their diet; *frugivores* for those species that depend on fruits or fruits and nectar for more than 50% of their diet; and *omnivores* for those species whose diet does not include any dominant food category. Foraging strata categories were *lower level* for those species that spend more than 65% of feeding time between ground and understorey strata; *higher level* for those species that spend more than 65% of feeding time between mid‐high and canopy strata; and *mixed level* for those species that spend less than 65% of feeding time in understorey or higher level strata. In addition, we used (iii) Kipp's distance, which is a wing morphological metric correlated with flight ability and dispersal. Birds with a shorter Kipp's distance have wings better suited for agile, manoeuvrable flight in more cluttered environments and for engaging in gleaning, hovering or quick darting to capture insects, fruit or other food items within confined spaces (Weeks et al., 2022); and (iv) body mass, which is a proxy of the pace of life and metabolic demands of the species.

### 2.3 Co‐occurrence network generation

We rely on a network approach, where bird species in both unlogged and logged forests are considered as nodes connected by edges, which represent significant associations among species. To include a temporal dimension, we analyse the community network in the two types of forests using abundance data collected over a period of 8 years. The inclusion of a temporal dimension is particularly relevant to assess if and how the network of co‐occurrences is influenced by the time elapsed from the last logging event compared to a stable old‐growth forest. Co‐occurrence networks, depicting the associations between birds within each plot, were generated using percolation networks. This approach is based on the identification of the critical value that describes the threshold similarity among pairs of species beyond which a network loses cohesion and becomes fragmented into disconnected clusters (Bellisario et al., 2019; Rozenfeld et al., 2008; Stauffer & Aharony, 1992).

For each sampling year and forest type, we first derived a similarity distance matrix of the shared co‐occurrences of species using the Bray‐Curtis index based on the number of captured individuals per plot. Then, starting from a fully connected network, we removed distances in decreasing order (i.e. most dissimilar species first), until the network reached the threshold value beyond which it becomes fragmented into disconnected clusters. Each time a distance value was removed from the network, species are redistributed into clusters of different sizes, from largest to smallest ones, until the critical threshold (i.e. distance value) is identified and the network becomes disconnected (for more information about percolation theory, refer to Stauffer & Aharony, 1992). We identified percolation networks using the function *perc.thr* implemented in the package ‘sidier’ (Muñoz Pajares, 2013) of R (R Core Team, 2024).

### 2.4 Modularity and null models

We used modularity (Q) to measure the pattern of aggregation/segregation of birds in logged and unlogged forests, respectively. Formally, Q measures the degree to which a network subdivides in densely connected groups of nodes (aka modules) with only sparser connections between groups (Newman & Girvan, 2004). High values of Q would indicate an overall tendency of networks to cluster into subgroups of species that have high spatial overlap, while the opposite is true for low values of Q. From an ecological point of view, a highly modular structure would suggest an increased pattern of segregation within the community, which is a situation likely associated with disturbance‐driven shifts in habitat‐use requirements or resource‐use partitioning (Garay‐Narváez et al., 2014; Valdovinos et al., 2009). We used the function *rnetcarto* in the ‘rnetcarto’ package of R (Doulcier & Stouffer, 2023) to compute modularity with simulated annealing.

To measure the extent to which the observed pattern of associations reflects non‐random associations among birds, we constructed 100 null models for each network by randomly reshuffling the raw observational data among plots (i.e. the corrected abundances) keeping the number of species (richness) and species incidence (i.e. the number of species observation *per* plot) constant. The significance of a modular partition for each network in each year was then tested by measuring whether the observed *Q* values (*Q*_obs_) were significantly higher or lower with respect to null models. We used the *z*‐score, *z* = (*Q*obs – *Q*_mean_)/*Q*_SD_, with *Q*_mean_ and *Q*_SD_ as the average and standard deviation of simulated *Q* following null models. Values of *z* > 2 and *z* < 2 indicate a significantly lower and higher modularity of networks compared to their randomised counterparts, respectively.

### 2.5 Partner fidelity

We defined partner fidelity as the tendency of a given bird species to always co‐occur with the same species in different years (Bellisario et al., 2023). The modular partition of networks (i.e. the assignment of individual species to a given module) was then used to quantify the frequency with which species tended to co‐occur over the years, by means of the normalised mutual information (nmi), a metric widely utilised for assessing the performance of community detection algorithms (Danon et al., 2005). We organised data in a species (rows) × module affiliation (columns) matrix (**M**), and we used the nmi to compare the degree of congruence between the rows of **M** (i.e. the module to which each species belonged in a given year), which enabled us to obtain a symmetric matrix that expresses the pairwise mutual information among species (nmi_M_). We used the row means of nmi_M_ to quantify the degree of fidelity among species (*f*_co_), where values close to 1 indicate the overall tendency of a given species to always cluster with the same species over the years (high fidelity) and vice versa.

### 2.6 Co‐occurrence fidelity and functional traits

To test whether the functional strategies of species influence the fidelity of co‐occurrence in different forest types, we employed beta regression models to relate *f*_co_ with functional traits. Beta regression is a technique well‐suited for modelling data that are limited to open intervals such as proportional data or measures derived from indices bounded between 0 and 1 (Douma & Weedon, 2019). This modelling approach avoids specific issues associated with bounded data, for example, in the case of non‐normal error term distributions and unequal variance. Formally, beta regression is similar to a GLM with which it shares the same components (the random and systematic components as well as the link function), but beta regression assumes that data can be modelled by a beta probability distribution (Douma & Weedon, 2019). We used the function *betareg* in the ‘betareg’ package of R (Cribari‐Neto & Zeileis, 2010) to regress the *f*_co_ values against the log‐transformed body mass and Kipp's distance, trophic niche and feeding strata.

### 2.7 Temporal changes in co‐occurrence

We examined the temporal variation in species associations to explore the effect of landscape transformation (logged vs. unlogged) on the restructuring of avian communities over time that could be masked by network metrics. Indeed, changes in co‐occurrence can be determined by a range of conditions able to influence the way with which associations are restructured among species as, for example, changes in spatial occupancy or population asynchrony among species pairs (Freilich et al., 2018; Twining et al., 2022). We used a simple linear model relating the temporal change in species abundance and co‐occurrence (measured by the species link density, which is the number of realised co‐occurrences that a species had from all possible ones) in both logged and unlogged forest (Kay et al., 2018). Changes were quantified by means of the Kendall τ coefficient, which is used to detect the presence of a monotonic tendency in the chronological series, and varying between −1 (negative, decreasing trend) and 1 (positive, increasing trend). We used the Kendall τ coefficient to provide a quantitative index of each species, independently from its statistical significance. This enabled us to determine whether those species experiencing a decline in terms of relative abundance were those losing co‐occurrences, and vice versa, and to classify species as having: (1) both increased co‐occurrence and abundance (‘Keystone connectors’ or ‘Dominant network hubs’); (2) reduced co‐occurrence but increased abundance (‘Opportunistic dominants’ or ‘Non‐interactive dominants’); (3) both reduced co‐occurrence and abundance (‘Decliner’ or ‘Ecological drift species’); and (4) increasing co‐occurrence but reduced abundance (‘Resilient key players’ or ‘Network stabilisers’).

## 3 RESULTS

### 3.1 Differences between old‐growth unlogged and logged forests in species co‐occurrences over time

**FIGURE 2:**
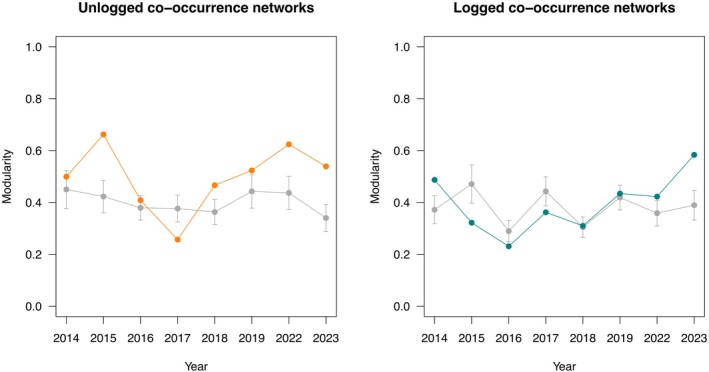
Trends in the modular structure (Q) of species co‐occurrence networks for unlogged and logged forests. Grey lines represent the average (±SD) simulated values of Q following null models (see main text). Note that there is an unequal interval in the caption because fieldwork was not permitted during the COVID‐19 years of 2020 and 2021.

Modularity was significantly different from the null model expectations in most of the study years (6 of 8 in both forest types). This outcome indicates a greater chance of species clustering in well‐defined groups or not clustering compared to a random distribution of species abundances (Figure 2). Modularity values were quite high (*Q*_log_ = 0.407 ± 0.111, *Q*_unlog_ = 0.497 ± 0.127), but did not differ between forest types (Student *t* = − 0.706, df = 7, *p* = 0.491), nor did they show any significant trends over the years (Mann–Kendall τ < 0.2 and *p* > 0.4 in both cases).

### 3.2 Patterns of fidelity between species

**FIGURE 3:**
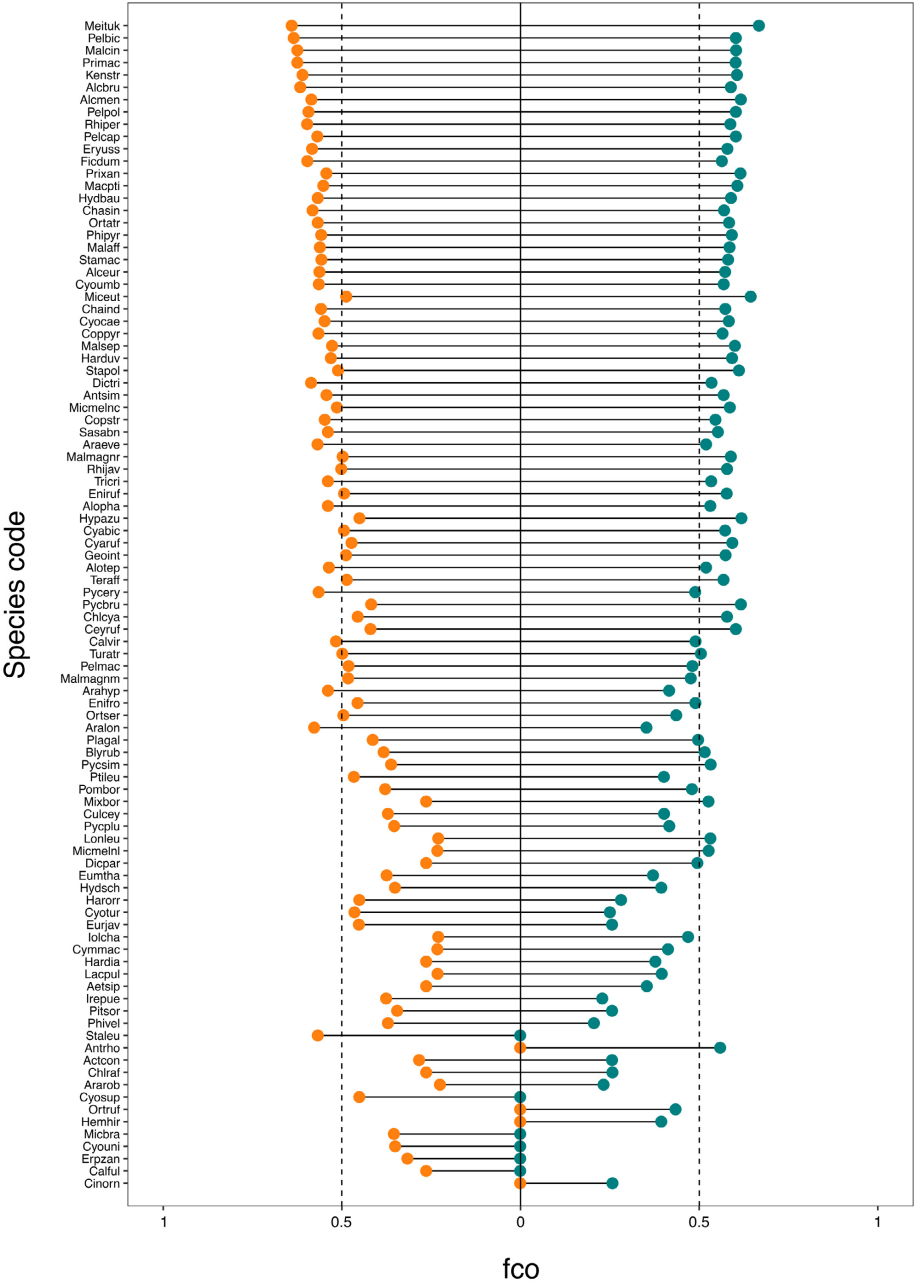
Lollipop charts showing the average fidelity score per species (*f*_co_) in both unlogged (orange points) and logged (green points) forest types. Points intersecting the zero line (solid line in figure) identify the absence of a given species in the corresponding forest portion. Values between 0 and 0.5 identify those species characterised by relatively low values of fidelity, while those having *f*_co_ > 0.5 can be considered as having high fidelity. Data in the graph are ordered following a decreasing order of cumulative *f*_co_ measured in both forests.

Unlogged and logged forests had similar values of co‐occurrence fidelity (i.e. the extent to which a given species always co‐occurs with a same given species over time). We found 43 and 56 of 95 species in unlogged and logged with high values of fidelity scores (>0.5), respectively (Figure 3). Some species (e.g. yellow‐breasted flowerpecker, Horsfield's babbler, chestnut‐rumped babbler, rufous piculet, striped‐wren babbler, scaly‐crowned babbler, Sunda fulvetta and spotted fantail) showed high values of fidelity in both types of forest. By contrast, other species (e.g. Sunda scimitar babbler, grey‐headed canary‐flycatcher, long‐billed spiderhunter and maroon‐breasted philentoma) had low values of fidelity in both types of forests.

Unlogged and logged forests also showed some subtle differences in the species co‐occurrence fidelity. Purple‐naped spiderhunters and little spiderhunters had higher average values of fidelity in unlogged than in logged forest (Figure 3), indicating a reduced fidelity in the logged forest. By contrast, rufous‐backed dwarf‐kingfisher, cream‐vented bulbul and white‐bellied munia had lower fidelity in unlogged than in logged forest (Figure 3). We detected a fair percentage of species (6 and 4 species in unlogged and logged forest, respectively) only in one of the forest types with varying values of *f*_co_; for example, white‐necked babbler, red‐throated sunbird and erpornis (Figure 3). Thus, we used *f*_co_ values in a beta‐regression model to test whether and how functional traits influence differences in co‐occurrence fidelity between forest types.

### 3.3 Influence of the functional traits on the species co‐occurrence

The results of the beta regression models showed that the propensity of species to be consistent in their pattern of co‐occurrence over the years mainly depends on trophic niche and Kipp's distance in both forest types (Table 1). Species with more specialised diets showed a significant tendency towards low fidelity; specifically, frugivores had lower fidelity in unlogged forests, while invertivores had low fidelity in both unlogged and logged forests. Species with large Kipp's distance values (i.e. species with long, pointed wings) showed a significant tendency to have low value of fidelity in both types of forest. Therefore, species that engage in foraging modes such as aerial foraging, soaring and gliding exhibited a higher tendency of avoiding other potentially interacting species.

**TABLE 1:** Influence of the functional traits on the species co‐occurrence in unlogged and logged tropical forests.

Predictors	Unlogged			Logged		
	Estimates	CI	*p*	Estimates	CI	*p*
(Intercept)	1.71	0.808–2.611	0.049	1.71	0.828–2.592	0.041
Log (body mass)	1.01	0.872–1.147	0.92	1	0.862–1.1372	0.994
Log (Kipp's distance)	0.76	0.603–0.916	**0.007**	0.81	0.653–0.966	**0.031**
Lower foraging stratum	0.92	0.763–1.076	0.313	0.89	0.752–1.027	0.175
Mixed foraging stratum	1.08	0.864–1.295	0.45	0.9	0.723–1.076	0.29
Frugivore	0.6	0.364–0.835	**0.01**	0.76	0.466–1.05	0.147
Invertivore	0.72	0.484–0.955	**0.048**	0.72	0.484–0.955	**0.05**
Omnivore	0.76	0.485–1.034	0.149	0.71	0.455–0.964	0.066
*n*	91			89		

*Note*: Bold values indicate significant *p*‐values.

### 3.4 Temporal changes in co‐occurrence

Birds in unlogged forest showed a similar trend in the changes of both annual abundances and co‐occurrences over the study period, indicating a positive association between them (Kendall *τ* −0.082 ± 0.296 and *τ* −0.09 ± 0.244, respectively). Conversely, birds in logged forest showed a slightly larger decrease in both annual abundance and number of co‐occurrences (Kendall *τ* −0.211 ± 0.320 and Kendall *τ* −0.098 ± 0.244, respectively). Overall, changes in abundance and co‐occurrence showed a significant relationship in both forest types, highlighting a general trend of increasing co‐occurrence with increasing abundance (adjusted *R* ^2^ > 0.3 and *p* < 0.001 in both cases).

**FIGURE 4:**
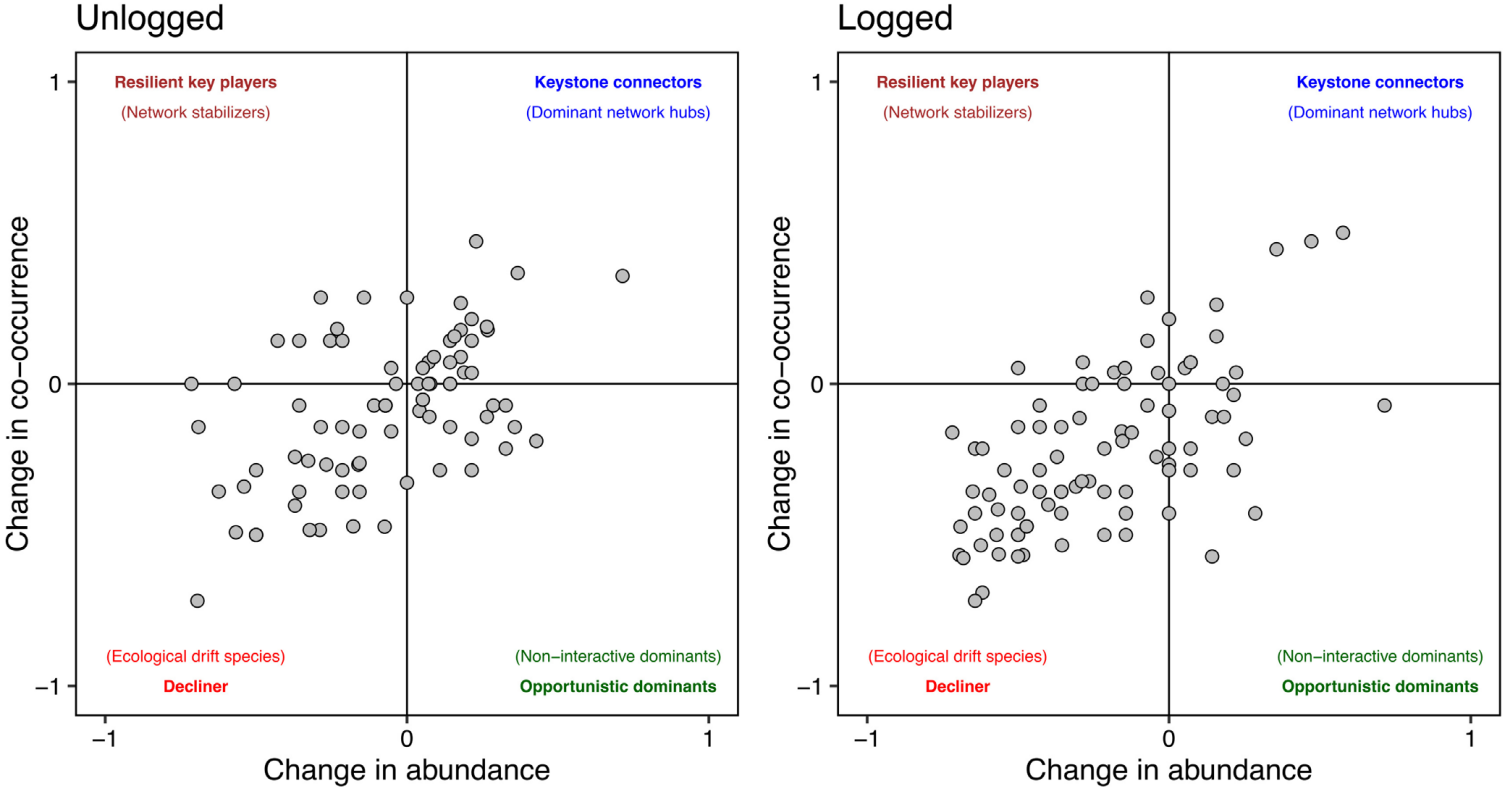
Relationships between temporal change in abundance and temporal change in co‐occurrence of species within unlogged and logged forest. Changes were quantified by means of the Kendall *τ* coefficient. Species included in this figure are reported in Table S1 with their values of both abundance and temporal changes.

Twenty‐seven species in unlogged forest and 13 in logged forest (seven of which are shared between forest types) had an increase in the number of co‐occurrences with the increase in abundance (‘dominant network hubs’, Figure 4). Similarly, 11 species in unlogged forest and 10 species in logged forest (three of which are shared between forest types) showed an increase in the number of co‐occurrences with a decrease in abundance (‘resilient key players’, Figure 4). Fifteen species in unlogged forest and 15 in logged forest (four of which are shared between forest types) had a decrease in co‐occurrence with an increase in abundance (‘opportunistic dominants’, Figure 4). Finally, 38 species in unlogged forest and 51 species in logged forest (23 of which are shared between forest types) showed a general decline in both abundance and co‐occurrence over the study period (‘decliner’, Figure 4).

When considering the role of single species in the bidimensional space provided by changes in abundance and co‐occurrence (Table S1), some species had different roles in the two types of forest, while others had similar roles. For example, little spiderhunter is classified as a ‘opportunistic dominant’ in the unlogged forest, but as a ‘resilient’ in logged forest, while Sunda scimitar‐babbler is classified as a ‘dominant network hub’ in the unlogged forest, but as a ‘decliner’ in the logged forest (Table S1).

## 4 DISCUSSION

We show novel evidence for the resilience of a tropical community of understorey birds to intense forest logging. However, forest logging influenced the patterns of co‐occurrence, and this was explained by two functional traits of species (trophic niche and Kipp's distance). We also found that the changes in co‐occurrence were significantly driven by changes in abundance in both types of forest.

The logged forest plots included in this study were logged intensively in 1970–1990 and again in 2001–2007 (Fisher et al., 2011). Although the last human disturbance occurred just 7 years before the start of our long‐term study, we did not find any evidence for a change of modularity over time, probably indicating that the community of understorey birds was resilient to selective logging. In logged, but not in unlogged, forests, we detected a non‐negligible deviation of the modularity score from the null model in the year 2016. The observed score was smaller than expected from the null model, which indicates that, in 2016, there was a change in the community structure, a situation that might be caused by changes in habitat‐use requirements or partitioning use of resources owing to some type of environmental disturbance. The climate of Borneo is strongly affected by El Niño, which causes a strong reduction of rainfall, particularly during the dry season (May–October; Aldrian & Dwi Susanto, 2003), and the weather during the field season of 2016 was particularly dry and hot, which might have amplified the effects of logging‐induced changes in habitat structure on the birds. Drying events may lead to changes in habitat structure (e.g. vegetation density), abiotic conditions (e.g. increasing temperatures) and food availability, with potential consequences for the animal communities (e.g. Bonal et al., 2016; Sherry, 2021). For example, studies on tropical birds found that droughts may influence their distribution, abundance and survival (e.g. Campos‐Cerqueira & Aide, 2021; Wolfe et al., 2025).

We found that two species of nectarinids, little spiderhunter and purple‐naped spiderhunter, had higher average values of fidelity in unlogged than in logged forest. These species are common in both types of forest, with little spiderhunter occurring in higher abundance in logged than in unlogged forests of Borneo (Costantini et al., 2016; Messina et al., 2021). Higher abundance in logged forests might explain the low fidelity in that type of forest because the species increases its niche overlap with other species. Spiderhunters are omnivorous and their diet includes spiders and other arthropods. A previous study showed that, in the understorey of logged forests, the abundance of spiders increased compared to unlogged forests (Edwards et al., 2011). Therefore, spiderhunters foraging in the understorey might be those more positively affected by changes in the arthropod communities. Our results also show that other species (rufous‐backed dwarf‐kingfisher, cream‐vented bulbul and white‐bellied munia) tended to co‐occur more frequently in logged than in unlogged forests, indicating that for these species, environmental filtering might be a relevant mechanism. This might be because these species tend to be more common in ecotonal zones of logged forests.

In this system, co‐occurrence of species with specialised diets (frugivores and invertivores) was particularly influenced by whether the forest was logged or unlogged. Previous work found that those species most adversely affected by logging were large‐bodied, ground‐feeding species with high trophic positions. In particular, babbler species (invertivores) were recorded in both forests as having significantly higher trophic positions in logged than in unlogged forests (Hamer et al., 2015). These high trophic positions were found mainly among terrestrial foragers, which might indicate a greater propensity to feed on predatory arthropods. These results suggest that, in logged forests, the patterns of co‐occurrence in species with more specialised diets might be determined by limiting similarity mechanisms through competition for shared food resources. This differential resilience of species with similar feeding habits to logging also points to the need for further understanding of optimal logging strategies that might reduce intra‐guild competition (Edwards, Magrach, et al., 2014).

The analyses of temporal changes in co‐occurrence and population abundance may help to infer about mechanisms regulating interactions between species and identify those species that are more vulnerable to selective logging. Our results show that temporal changes in co‐occurrence were significantly affected by changes in local abundance, but also that there was much variation unexplained by abundance, indicating that other factors need to be considered and explored in future studies. In some species, an increase in local abundance is associated with an increase in co‐occurrence, suggesting that these species probably increased their interactions or simply tend to occupy the same forest plots. For example, this is the case of leaf‐litter babblers and Bornean ground‐babblers in the unlogged forest, suggesting that pristine forests may reduce competition between them. Further studies are needed to assess this hypothesis.

Species increasing in both local abundance and co‐occurrence over the study period might be keystone connectors or dominant network hubs that play a crucial role in maintaining the structure and function of the ecological network. Previous work showed that birds of prey are typical keystone species because of the direct pressure they impose on prey that indirectly may cascade through the entire community (Burgas et al., 2021). Our dataset did not include any predators; thus, our keystone species might have influenced the ecological network through other routes, such as their competitive dominance, behavioural responses to forest changes or changes in diet (García‐Navas et al., 2024; Rahman & Candolin, 2022) that need to be explored in future studies. For example, we found that Bornean ground‐babblers and Bornean banded pitta were keystone connectors in logged forests (Figure 4). One explanation for this result might lie in the foraging niche of these species because Bornean ground‐babblers and Bornean banded pitta are ground invertivores. It might be that the natural recovery of the forest increased diversity of arthropods, restoring dietary conditions for both species, thus favouring their increase in abundance and co‐occurrence as a result. Under these circumstances, these species might be keystone connectors of the (sub‐) community of ground‐feeding birds. Further studies are needed to test this hypothesis.

We also observed species that increase in abundance, but decrease in co‐occurrence, indicating that they become numerically dominant in a forest but engaged in fewer or less significant ecological interactions (defined as opportunistic dominants, abundant isolates or non‐interactive dominants, depending on their ecological context). One reason for this result might lie in the niche theory, which states that niche segregation would occur to reduce competition for similar resources between some species that live in sympatry (Basile et al., 2021; Hadly et al., 2009; Kosicki, 2022; MacArthur & Levins, 1967; Mansor & Ramli, 2017). This might be the case for sooty‐capped babblers and scaly‐crowned babblers, which are both invertivores feeding between the understorey and mid‐storey strata of the forest, that were opportunistic dominants in both forest types. Furthermore, our results show that rufous‐collared kingfisher was an opportunistic dominant in unlogged forests and a keystone connector in logged forests, while the reverse occurred in grey‐chested jungle‐flycatchers and rufous‐chested flycatchers. We do not know the reasons of these differences between forest types because there is limited information on the behavioural interactions between different understorey bird species. Recording of species interactions in both forest types is an important area for future research to elucidate the mechanisms driving the co‐occurrence patterns.

Conversely to opportunistic dominants, some species show an opposite pattern: They decrease in abundance but increase in co‐occurrence (resilient key species or network stabilisers), indicating that they become more central or critical in the ecological network, despite a decline in population size. These species may link otherwise disconnected parts of the network and contribute to ecosystem resilience despite their rarity. For example, some species can maintain high network connectivity through mixed‐species flocking behaviour, where flock leaders can create stable networks even as their abundance declines due to habitat fragmentation (Jones & Robinson, 2021). These species could be at risk of extinction if population decline continues, which might have dramatic cascading effects on the community assemblage. Thus, they might represent key targets of conservation efforts.

Conversely to resilient key species, other species that decrease in abundance over time within a type of forest also had a reduced co‐occurrence (decliner), suggesting a drastic reduction in their contribution to the resilience of the understorey community. Most of the decliners are species spending variable amounts of time feeding between the understorey and higher strata, such as bulbuls. Their lower abundance associated with reduced co‐occurrence in the understorey might be driven by changes in feeding habits due to the mean height of fruiting trees. There are resilient key species that become decliners in the logged forest (e.g. Horsfield's babbler, black‐throated wren babbler and Asian emerald dove). These species might be more vulnerable to abiotic and biotic changes in the forest and at risk of isolation in the ecological network as a result. Thus, they might be the species that benefit most from designating programmes for their conservation (Barnes et al., 2023). Previous work suggested that the impact of rare bird species on the overall stability of avian communities in relation to disturbances might be low compared to that of more common species (Angeler et al., 2022). However, our results suggest that not all rare species play similar roles in the community; thus, it is important in future work to address the differences between those rare species that are somehow resilient and those that decline.

## 5 CONCLUSIONS

The community of understorey birds was resilient to forest logging, although some guilds and single species were more sensitive, resulting in significant changes in their co‐occurrence with other species within the ecological network. Changes in local abundance influenced patterns of co‐occurrence, but also trophic niche and Kipp's distance had a significant effect on the response of birds to forest logging. Although many species that increased (or decreased) in abundance over the study period were also those that increased (or decreased) their co‐occurrence, some species did not follow this pattern. Thus, niche segregation and environmental filtering might operate concurrently to shape the community assemblage. Moreover, we found that, while some rare species increased their co‐occurrence, others did not. Thus, the proposed negligible role of rare species in the stability of avian communities might not be generalisable. Further work will be needed to delve further into the mechanisms that regulate species interactions (e.g. movement patterns) and the role of single species in the regulation of avian assemblages. Finally, our results point to the conservation potential of these logged forests because the habitat conditions present still favoured the resilience of the bird community, even when the forests were not subjected to restoration interventions.

## AUTHOR CONTRIBUTIONS

David Costantini and David P. Edwards conceptualised the study, with David P. Edwards, Suzanne Tomassi and Suzan Benedick coordinating different phases of the study; David Costantini, Simone Messina, Julia Weiss, Suzanne Tomassi, Cindy C. P. Cosset and David Edwards collected samples; and Bruno Bellisario analysed the data. David Costantini led the writing of the manuscript, with all authors contributing critically to the draft and giving final approval for publication.

## FUNDING INFORMATION

We thank the Italian Ministry for University and Research for a post‐doctoral fellowship awarded to S.M. (NextGenerationEU—Young Researcher ID: SOE_0000189), Gates Cambridge for a PhD studentship awarded to J.W., the Leverhulme Centre for Advanced Biological Modelling for a PhD studentship awarded to C.C.P.C., the Rufford Foundation for funding 2 years of fieldwork and NERC awarded to D.P.E. (NE/W003708/2 and NE/Z50404X/1).

## CONFLICT OF INTEREST STATEMENT

The authors declare no conflicts of interest.

## PERMITS

All experimental procedures were approved by the Sabah Biodiversity Council (access licence number: JKM/MBS.1000‐2/2 JLD.6(39), JKM/MBS.1000‐2/2 JLD.7(57) and JKM/MBS.1000‐2/2 JLD.16(132)).

## Supporting information


**Table S1.** List of species, functional traits and data used for statistical analyses.

## Data Availability

Data included in this study are available at Figshare: https://doi.org/10.6084/m9.figshare.29163536 (Costantini et al., 2025).
